# Factors related to monitoring during admission of acute patients

**DOI:** 10.1007/s10877-016-9876-y

**Published:** 2016-04-12

**Authors:** Thomas Schmidt, Camilla N. Bech, Mikkel Brabrand, Uffe Kock Wiil, Annmarie Lassen

**Affiliations:** 1 The Maersk Mc-Kinney Moeller Institute, University of Southern Denmark, Campusvej 55, 5230 Odense, Denmark; 2 Department of Emergency Medicine, Odense University Hospital, Odense, Denmark; 3 Department of Emergency Medicine, Hospital of South West Jutland, Esbjerg, Denmark

**Keywords:** Emergency departments, Computerized decision support, Patient monitoring

## Abstract

Understanding the use of patient monitoring systems in emergency and acute facilities may help to identify reasons for failure to identify risk patients in these settings. Hence, we investigate factors related to the utilization of automated monitoring for patients admitted to an acute admission unit by introducing monitor load as the proportion between monitored time and length of stay. A cohort study of patients admitted and registered to patient monitors in the period from 10/10/2013 to 1/10/2014 at the acute admission unit of Odense University Hospital in Denmark. Admissions with at least one measurement were analyzed using quantile regression by looking at the impact of distance from nursing office, number of concurrent patients, wing type (medical/surgical), age, sex, comorbidities, and severity conditioned on how much patients were monitored during their admissions. We registered 11,848 admissions, of which we were able to link patient monitor readings to 3149 (26.6 %) with 50 % being monitored <1.4 % of total admission time. Distance from nursing office had little influence on patients monitored <10 % of their admission time. But for other patients, being positioned further away from the office reduced the level of monitoring. Higher levels of severity were related to higher degrees of monitoring, but being admitted to the surgical wing reduce how much patients were monitored, and periods with many concurrent patients lead to a small increase in monitoring. We found a significant variation concerning how much patients were monitored during admission to an acute admission unit. Our results point to potential patient safety improvements in clinical procedures, and advocate an awareness of how patient monitoring systems are utilized.

**Electronic supplementary material:**

The online version of this article (doi:10.1007/s10877-016-9876-y) contains supplementary material, which is available to authorized users.

## Background

Patients of all sorts and with a wide range of diagnoses are treated in emergency departments (ED) around the world every single day. Keeping track of such a diverse group of patients challenges both clinicians and systems. To cope with this, several health information systems have been developed specifically for managing the flow and treatment of patients. Still, a substantial number of acutely admitted patients deteriorate during their admission with an increased risk of adverse outcomes [1]. There is widespread consensus that the risk of such deterioration can be reduced by a more frequent and rigorous approach to monitoring of patient vital signs [2]. However, the decision to continuously monitor a patient’s vital signs can still be a result of multiple causes; e.g., raised patient concern, or to optimize working procedures by not having to attach sensors repeatedly on patients requiring frequent registrations. Or perhaps also as a mean for boosting situational awareness in high load periods [3]. As such, monitoring can be viewed as an important part of the afferent limb as it provides feedback needed to initiate interventions [4]. The notion that an increased rate of vital sign registrations reduce the risk of adverse events has spurred a surge in quality assurance programs worldwide, despite concerns about the effectiveness of routinely measured vital signs have been raised [5]. Partly because the process of vital sign registrations is associated with both human and machine related errors [6]. Evidently, there exists a gap between the clinical reality and the vital sign registration procedures defined by guidelines [7, 8], and as most research on automated monitoring has been conducted in the settings of intensive care units (ICU) [9] we in this work focus instead on monitoring in acute settings.

We expect very sick patients to be monitored more than the less sick; and, it has been documented that clinicians are prone to skip vital sign registrations of less severe patients [8]. This can potentially lead to dire consequences for these patients as the risk of deterioration is present across all severity levels [10]. Understanding the utilization of patient monitoring systems in the dispersed and shared working environments of EDs and acute wards may help to identify some of the reasons for failure to rescue patients [11].

Although increased levels of automated monitoring may improve the detection of patient deterioration, several factors may influence the extent to which a patient is being monitored. The purpose of this paper is to investigate the use of automated monitoring of patients admitted to an acute admission unit by analyzing how much the effects of distance from the nursing office, number of concurrently admitted patients, wing type (medical/surgical), age, sex, comorbidities, and severity change conditioned on how much patients are monitored during admission.

## Methods

Our work is based on a cohort study conducted at the acute admission unit at Odense University Hospital, a 1000 bed teaching hospital serving as a primary hospital for a local population of 280,000 citizens. After initial assessment in the ED, admitted patients projected for short-term stays of up to 48 h are transferred to the admission unit. Patients can be transferred to intensive care on clinical indication. If deemed necessary, an intensive care consultant is contacted and need and relevancy for transfer has to be acknowledged by both parties. The ward is structured into three wings, one wing for surgical patients (12 beds), and two wings for medical patients (18 and 16 beds).

The processing and management of patients in this ED has been documented in an earlier field study conducted by the first author [12]. In relevance to this paper, the most important aspects are the department’s reliance on a 5-level triage and observation regimen system which defines a baseline level of clinical alertness for each level (Blue, Green, Yellow, Orange, Red), and that the bedside ward is structured into three distinct wings, with a nurse office in the center of each wing. Each bed on every wing is equipped with its own vital signs monitoring unit. The degree of monitoring for each patient is defined by the attending physician based on the observation regimen, and in some cases adjusted by nurses afterwards. The assigned observation regimen is registered in the patient’s electronic medical record.

### Data description

All vital signs from all monitors at the ward in the period from 10th of October 2013 to 1st of October 2014 are captured in a research database using a customized application written in Java. The department relies on Philips IntelliVue MP30/50 monitors in a networked setup as monitoring information from beds are aggregated on Philips IntelliVue Information Centers in each nursing office. When a patient is attached to a monitor our system receives a packet containing vital sign information at different intervals. Every minute we register heart rate and respiration rate from 3-lead ECG, pulse rate and peripheral oxygen saturation (spO2) measured via pulse oximetry. Depending on the clinical assessment of the patient, systolic and diastolic blood pressures are registered in intervals from 5 to 60 min using cuffs. In this project, nurses are asked to enter patient identification into the Philips monitors by personal identification number (PIN) and name, thus enabling us to link the vital signs from a given bed location to a specific patient. Apart from this, the data collection instills no further change to existing clinical practice. We include all patients registered on the monitor with at least one measurement. However, not all patients get their information entered into the monitors, and consequently our system holds an amount of vital values which we cannot associate with specific patients. The characteristics of the not-identified patients are included in our analysis to enable between-group comparisons.

Using the PIN, we link the collected data with supplemental information from population based national patient registries. Arrival, admission, and discharge information are retrieved from the Danish National Patient Registry [13, 14].

### Analysis

We aim to describe patient and department related factors and their relationship to how much patients are monitored. During their admission, patients will be intermittently attached to bedside vital sign monitors. We use the extent to which a patient is monitored as our point of interest by defining *monitor load* as the proportion between time attached to monitor and length of stay. A monitor load percentage of 100 % means that the patient is being continuously monitored throughout their entire admission; which in the study settings translates to 1 automatic reading per minute.

Table 1 provides an overview of the exposure variables used in the model. Categorical variables are automatically converted to dummy variables. Concurrent patient load is calculated based on the number of active beds in the wing during each patient’s admission period. The analysis includes exposures relating specifically to each individual; age, observation regimen, Charlson comorbidity index [15], and sex. And external factors; distance from nursing office, concurrent load, and wing type. The relationship between monitor load and each of the exposure variables are investigated via scatter or box-plots. We focus specifically on the relationship between distance from nursing office and monitor load using univariate linear regression analysis, and investigate how the relationship between these variables change conditioned on what quantile of monitor load we look at. All variables are combined in a multivariate model to examine the partial effects of each variable when controlling for all others [17–19]. We apply QR for the quantiles τ = (0.10, 0.25, 0.50, 0.75, 0.90) and linear multiple Ordinary Least Squares regression.

**Table 1:** Overview of exposure variables

Independent variable	Type	Values	Description
Comorbidity Index (CI) [15]	Ordinal	A, B, C, D	A: CI = 0, B: CI = 1; C: CI = 2; D: CI > 2
Severity	Ordinal	Regimen levels (1–5)	See [16]
Age	Ordinal	15 − x	
Sex	Nominal	Female/male	
Distance	Ordinal	0 − x	Distance in meters from office on each wing
Wings	Nominal	MAU1, Surgial, MAU2	MAU1-2: (Medical Admission Unit) wings
Concurrent load	Ordinal	1 − x	Average number of patients admitted to the wing per day during the admission period of the patient

We correct for multiplicity using the Holm–Bonferroni method, and investigate issues with multicollinarity between exposure variables using the variance inflation factor (VIF) [20]. Finally, we test for differences in regression coefficients between the quantiles using the ANOVA method.

Between group comparison for distribution of triage categories as severity, and comorbidities between patients registered on the monitors, and not-registered patients are evaluated using Chi squared tests.

The preprocessing and regression analysis is conducted in R (version 3.1.1) using the quantreg package [21]. The data is preprocessed by calculating the all the aggregated exposure variables such as distance, comorbidity and concurrent load.

Access to the registry of patient data was approved by the Danish Data Protection Agency (Datatilsynet—J.nr. 2013-41-2238), and the Danish Health and Medicines Authority (Sundhedsstyrelsen—J.nr. 3-3013-518/1). The study has been presented to the Research Ethics Committee of Southern Denmark, but as this is a non-interventional study an approval was not needed according to Danish law.

## Results

During the data collection period there were 11,848 admissions to the acute ward representing 35,727 days. Of these we are able to link monitor use to 3149 admissions (26.6 %) for 10,844 days (30.4 %), representing 1031 fully monitored days. Patient monitor utilization was also registered for patients who we could not identify on their monitors, equating to 1271 fully monitored days. Patients in our dataset are on average admitted to the ward for 3.3 days, compared to 2.9 days for not-included patients. 115 of the patients admitted to monitors in the dataset experienced respiratory distress, seven patients suffered strokes, and one patient had both respiratory and heart related deterioration during admission. Table 2 summarizes data for patients identifiable from the monitors, and from patients not registered to monitors.

**Table 2:** Exposure characteristics

	Admitted to monitor	Not admitted to monitor
Number of admissions	3149	8699
Number of patients	2815	4104
Male [n (%)]	1526 (48.4)	4314 (49.6)
Mean age		
Male	63.8 years, SD = 18.5 years	60.9 years, SD = 21.1 years
Female	66.8 years, SD = 20.9 years	63.8 years, SD = 22.6 years
Comorbidity (Charlson Score (CS)) [n (%)]		
(A) CS = 0	1124 (35.7)	3481 (40.0)
(B) CS = 1	641 (20.3)	1643 (18.9)
(C) CS = 2	498 (15.8)	1297 (14.9)
(D) CS > 2	886 (28.2)	2278 (26.2)
Triage [n (%)]		
Missing	514 (16.4)	1554 (17.9)
Blue	7 (0.2)	27 (0.3)
Green	431 (13.7)	1341 (15.4)
Yellow	1301 (41.3)	3333 (38.3)
Orange	842 (26.7)	2315 (26.6)
Red	54 (1.7)	129 (1.5)
Average number of registered vital signs/admission	408 registrations, SD = 633	–
Wing [n (%)]		
Surgical	809 (25.7)	3948 (45.4)
MAU1	1015 (32,2)	4751 (54.6) [both medical wings]
MAU2	1325 (42.1)	

While the differences in proportions for both comorbidities and triage between patients identifiable on the monitors, and other patients, are statistically significant, there are no substantial clinical differences between these factors. We do however observe that a lower percentage of surgical patients are identifiable on the monitors.

**Fig. 1:**
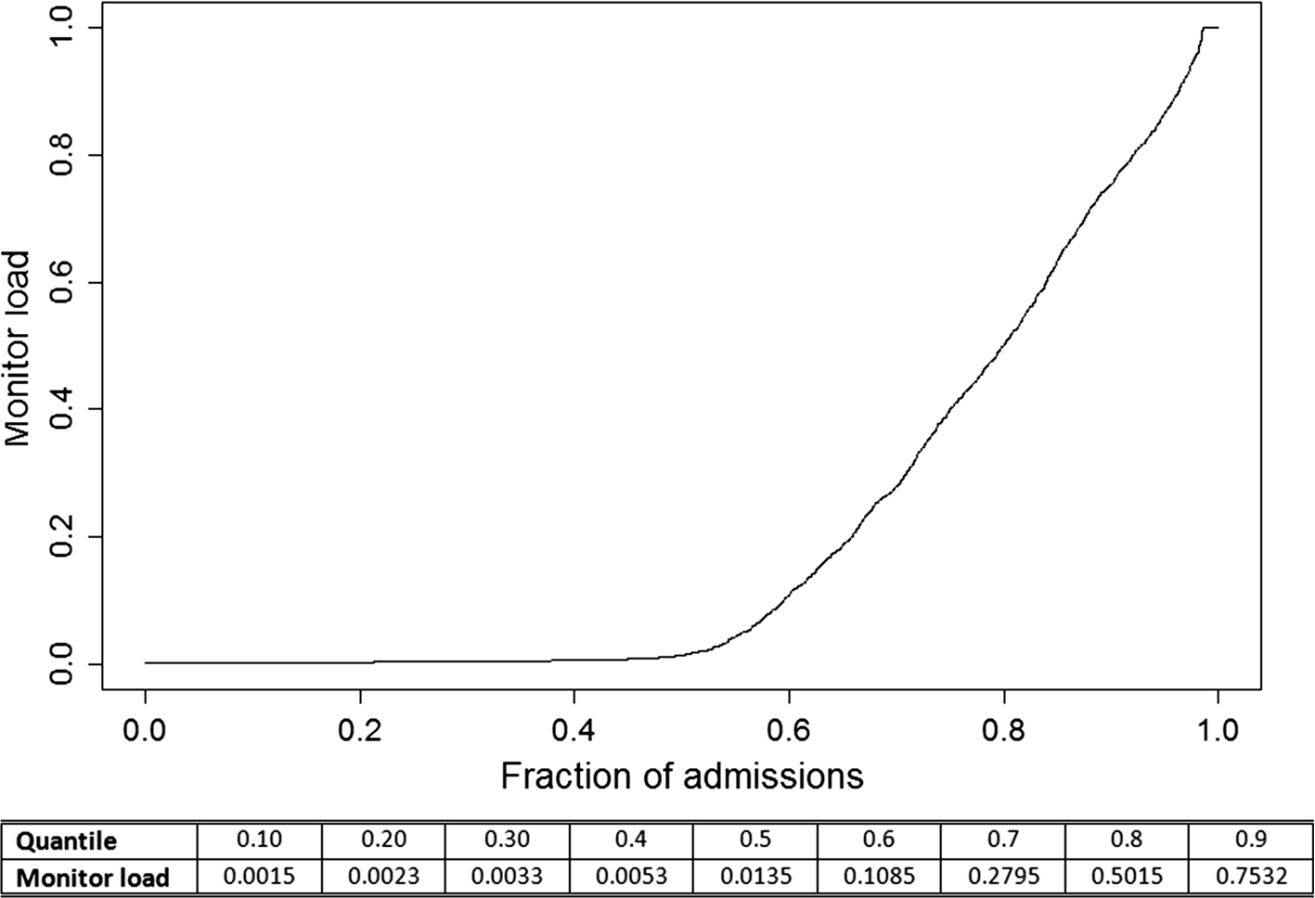
Quantile plot for the response variable—illustrating the distribution of monitor load by its quantile distribution

In Fig. 1, we observe the highly skewed distribution of how much patients are monitored. 50 percent of all the admissions have a monitor load of less than 0.014; meaning that half of all the cases are monitored less than 1.4 percent of their admission. Moving upwards, 70 percent of all admissions are monitored less than 28 percent of their total admission length. Thus, as the distribution of monitor load is heavily right skewed, standard Ordinary Least Squares regression cannot provide plausible insight. However, applying a QR approach enables us to analyze the relationship between the different exposure variables and monitor load conditioned on monitor load.

**Fig. 2:**
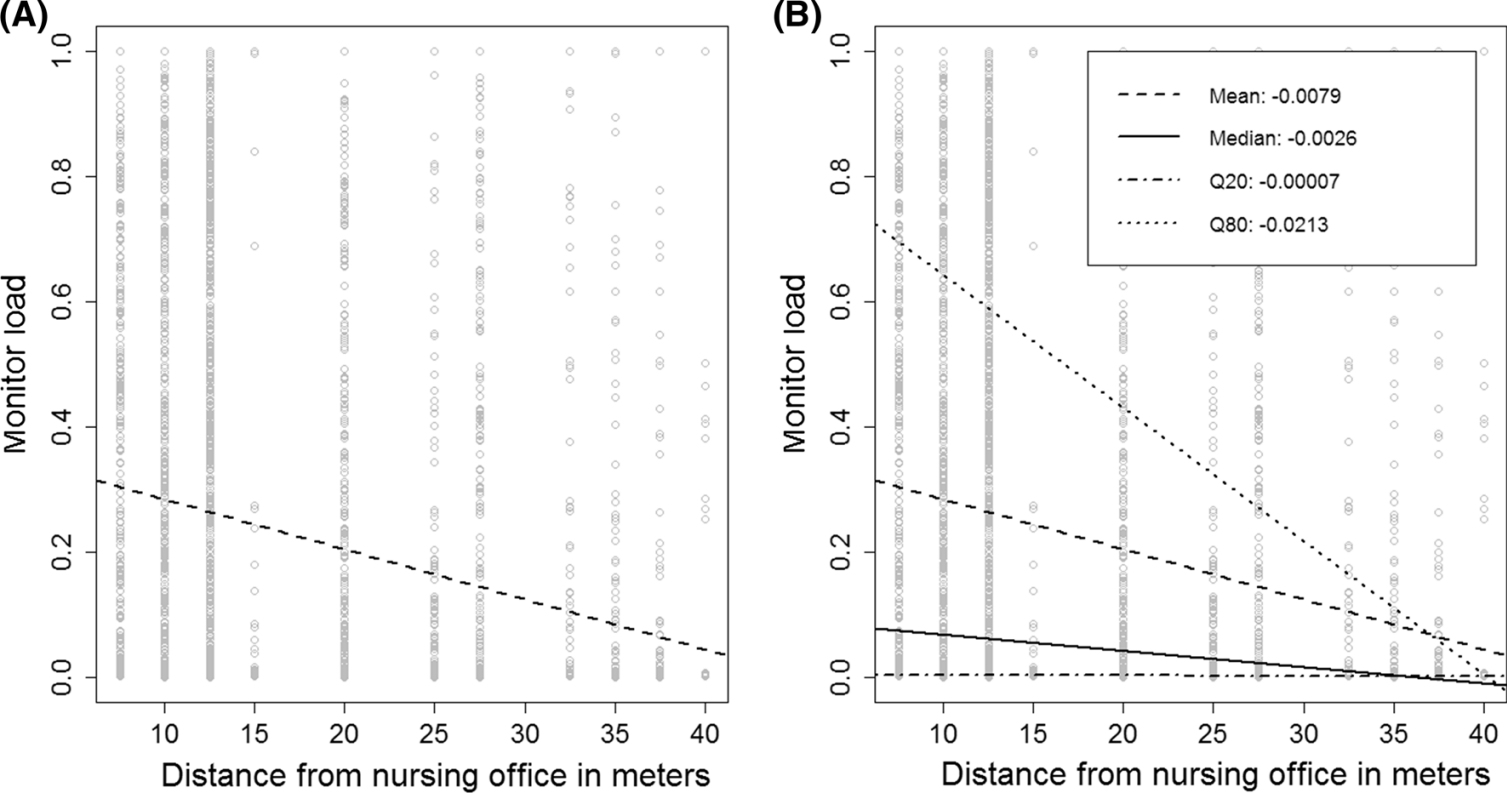
Univariate regression plot of Distance from nursing office and registered Monitor load. **a** Ordinary Least Squares (mean ased) linear regression. **b** Mean linear regression, Median (Q50), 20th Quantile and 80th Quantile linear regression

Figure 2 exemplifies this by showing the linear regression line of the relationship between distance from nursing office and monitor load in Fig. 2a, and quantile regression lines based on the 0.20, 0.50 (the median) and 0.80 quantiles in Fig. 2b. From the regression coefficients, we observe that the association between monitor load and distance from nursing office grows stronger for the upper quartiles of monitor load. Online Supplement 1 (Figure 4) shows the individual relationships between each exposure variable (age, sex, comorbidity group, triage, wing type, and the number of other patients treated during admission) and monitor load.

**Fig. 3:**
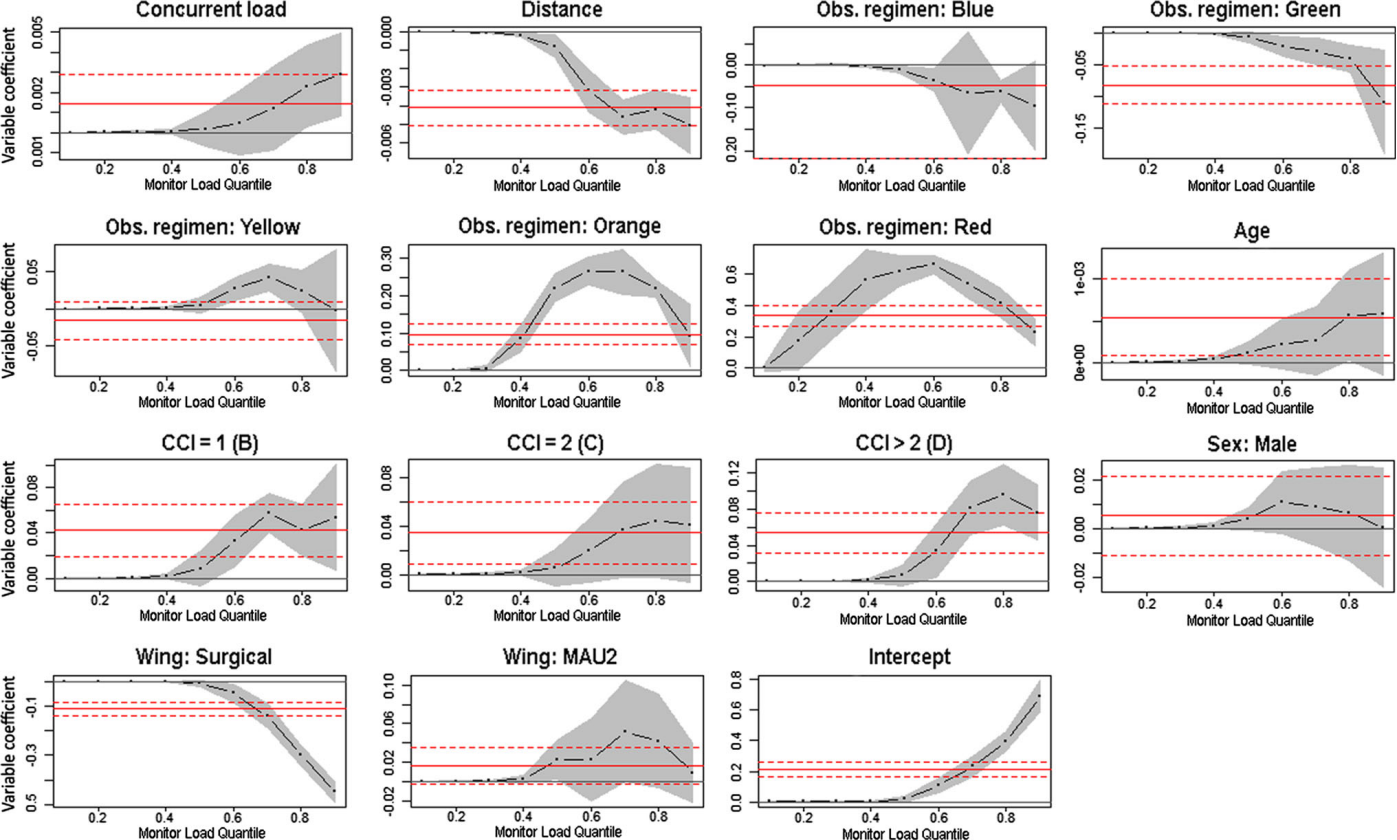
Quantile regression process plots for exposures—showing the regression coefficients for the quantiles of exposure variables and the intercept when controlling for all factors

The results of the multivariate QR results are shown for all exposures in Fig. 3 as quantile process plots from the 0.10th up to the 0.90th quantile. The solid horizontal line for each variable indicates the Ordinary Least Squares regression coefficient, and the dotted horizontal lines show the confidence interval. Similarly the QR regression results at each quartile are marked with the regression coefficient of the exposure variable, and the confidence interval as the grey band. E.g., we find that distance from nursing office has the strongest influence for patients who are monitored a lot (i.e., admissions in the upper quantiles of Fig. 1). For observation regimens, we find that Orange classes have a stronger influence across the quantiles of monitoring load, but also that its impact decreases for highly monitored patients.

Table 3 in Online Supplement 2 conveys the results of both regression approaches. Our multiple linear regression model has an adjusted R^2^ of 0.1719, and are thus comparable to those of [22], and the model is overall statistically significant. The VIF is below 1.62 for all exposure variables, and we thus dismiss issues of multicollinarity. The Holm–Bonferroni adjustment changes the significance of several exposure variables, but the ANOVA finds that all QR coefficients are significantly different from one another.

An example of how to interpret the results from Table 3 in Online Supplement 2 and Fig. 3 is provided in Online Supplement 3.

### Sensitivity analysis

To address and investigate the potential impact of missing values in the dataset, we reran the analysis with missing values removed. This had little impact on the distribution of the remaining triage coefficients, and did not substantially alter the exposure coefficients or their significance.

## Discussion

We find that distance from nursing office has little influence on patients monitored less than 10 % of their admission time. But for other patients who are monitored more than this, distance from nursing office becomes has more impact in reducing the degree of monitoring. We also note that higher levels of observation regimens have a significant impact on monitoring load. Being admitted to the surgical wing greatly reduces how much patients are monitored, and periods with a high amount of concurrent patients have little effect on the degree of monitoring.

The increased focus on identification of deteriorating patients can be seen in the body of published work on Early Warning Scores [23], Track & Trigger systems [24, 25], and Rapid Response Teams [26]. Although few of the existing deterioration detection systems in use have been rigidly validated [27, 28], the need to identify efficient means for keeping an eye on multiple patients is evident as the pressure on EDs is ever increasing. However, simply decreeing more monitoring of patients, does not necessarily reduce the proportion of patients with adverse events [29]. Vital sign readings are often used to support clinical intuitive hunches, and less as objective points of Ref. [30]. Even so, little research on what determine frequency of vital sign registrations have been published [22]. Since most assessment systems rely on intermittent or spot-driven observations, continuous monitoring in its current state may simply yield excessive amounts of data which can only be utilized fully through integration into clinical decision support systems. Also, the risk of more monitoring leading to alarm fatigue and habituation has to be factored in by careful consideration of calibrating the alarm thresholds [31, 32].

Recent studies have rectified the assumption that deviance from protocol is solely due to clinical misjudgments, and instead taken a more holistic approach to the problem by investigating several factors such as day of week, time of day, and characteristics of both patients and clinicians [33, 34]. In this study, we find evidence for adherence to observation regimen protocols through insight into how much patients are actually monitored during admission. Along these lines it is problematic that patients on the surgical wing are monitored much less than medical wing patients given that adverse events are also associated with post-surgical situations [35, 36]. This is probably a combined effect of differences in working procedures, culture between specialties as mobilization of post-surgical patients is considered important by surgical nurses, and the fact that many pre-surgical patients are unaffected until surgery, and that many orthopedic patients are admitted with minor surgical problems.

Quantifying the extent to which a patient is being monitored, may be an aid to bridge the current gap between usage of automated and manual monitoring as clinical work will continue to depend on tacit knowledge and intuition [37, 38]. Since the use of monitoring is increasing in all types of hospital departments, and as technology becomes more pervasive, the insight from this paper may provide guidance for system designers and clinicians a like.

Cabled monitoring as found in the settings of this study has several downsides; immobilization of patients, patient induced stress due to perceived severity, and loss of data during out of bed activities [39]. Consequently, much research effort has been put into the potential of wireless monitoring, but several practical obstacles such as battery life and poor communication networks still persist [40–42]. However, given that wireless monitoring could support temporary storage of vital signs on the device, would enable a smoother transition between hospital departments and reduce loss of information in out-of-bed periods. In this scenario, all patients could achieve a monitoring load of 100 %, thereby enabling more complete representation of their states and trajectories.

Interestingly, the decreasing impact of the most influential coefficients in our statistical analyses for patients who are continuously monitored, indicate that factors not included in our model prompt higher degrees of monitoring. Seeking to capture the complexity of patient monitoring in just seven exposure variables yields a very simplified model at best, and shows that patient monitoring is a complex and subjective endeavor. In this perspective, it would be interesting to include staff specific features such as clinical experience, department seniority, team composition, and clinical concern in future work. An important aspect we intentionally left out of the analysis is temporal influences. As both clinical work, and the vital signs of patients follow a circadian rhythm, these aspects may reveal valuable insight for the evaluation of existing clinical protocols.

## Limitations

This study was influenced by a number of limitations. The most important being our limited ability to link monitor utilization to specific patients, thus the study only includes patients deliberately registered to the bedside monitors by nurses. The percentage of patients who were identifiable by the monitors was highest in the early phases the data acquisition stage, and then gradually decreased. The monitor registration identification eventually plateaued, indicating that a dedicated subset of nurses persisted in registering patients to the vital sign monitors for us. This naturally induces a permutation of selection bias that is difficult to overcome in this kind of project. This selection bias is also evident as identifiable patients are slightly older, have longer hospital stays, are sicker, and are deemed in need of more frequent observations (Table 2). Although, the identifiable admissions in our analysis only account for 27 % of the total admissions in the entire period, the linked vital signs account for 45 % of all vital signs registered in the same period. This may either be a sign of issues with linking the vital signs accurately to admissions, but is also likely a seasonal indicator as the first 6 months had the highest inclusion rate, and took place during Q4-2013 till Q1-2014.

Another limitation is missing data, and inaccurate date and time values in the coupled registries. Issues with timestamps in data retrieved from Patient Administration Systems are well known in the scientific community. Also, the observation regimen classes originate from the triage classes assigned at arrival time, generally there is a direct mapping between triage and observation regimen for patients admitted to the acute admission unit, but not necessarily for all admissions. Finally, external validity of our findings may be challenged by the single site nature of our study. Yet, assessing the monitor load of patients may be of value to similar studies, and the design of future patient monitoring systems.

## Conclusion

As expected, there is significant variation concerning the how much patients are monitored during their admission to an acute admission unit, but the effect of the investigated factors varies depending on how much patients are monitored. We confirm that patients assigned to more severe observation regimen categories, are monitored more, but also show that both distance from the wing’s nursing office influence monitoring for most patients. Number of simultaneously admitted patients has a small effect across all levels of monitoring. Finally, we find a big difference between the extent to which monitoring is utilized at medical and surgical wings.

The results point to potential improvements in clinical procedures, and advocate an awareness of how patient monitoring systems are utilized. Formalizing the extent of monitoring can be utilized to assess the reliability of data from patients, and as a metric for expressing severity and clinical concern. The relationship between monitor load and patient specific outcomes such as medical emergency team activation or mortality is left for future studies to examine.

## Electronic supplementary material

Below is the link to the electronic supplementary material.

ONLINE SUPPLEMENT 1 – Fig. 4 Univariate plots of the relationship between monitor load and: (A) Patient age, (B) Triage (severity), (C) Charlson comorbidity index, (D) Sex, (E) Wings, (F) Number of concurrent patients admitted to the wing during each admission (TIFF 8610 kb)

ONLINE SUPPLEMENT 2 – Table 3 Regression results (DOCX 33 kb)

ONLINE SUPPLEMENT 3 Guide to interpretation of quantile regression results (DOCX 14 kb)
