# Supplementary figures and images for: Factors related to monitoring during admission of acute patients
Source: J Clin Monit Comput. 2016 Apr 12;31(3):641–9. doi: 10.1007/s10877-016-9876-y (PMC5403848; doi:10.1007/s10877-016-9876-y)

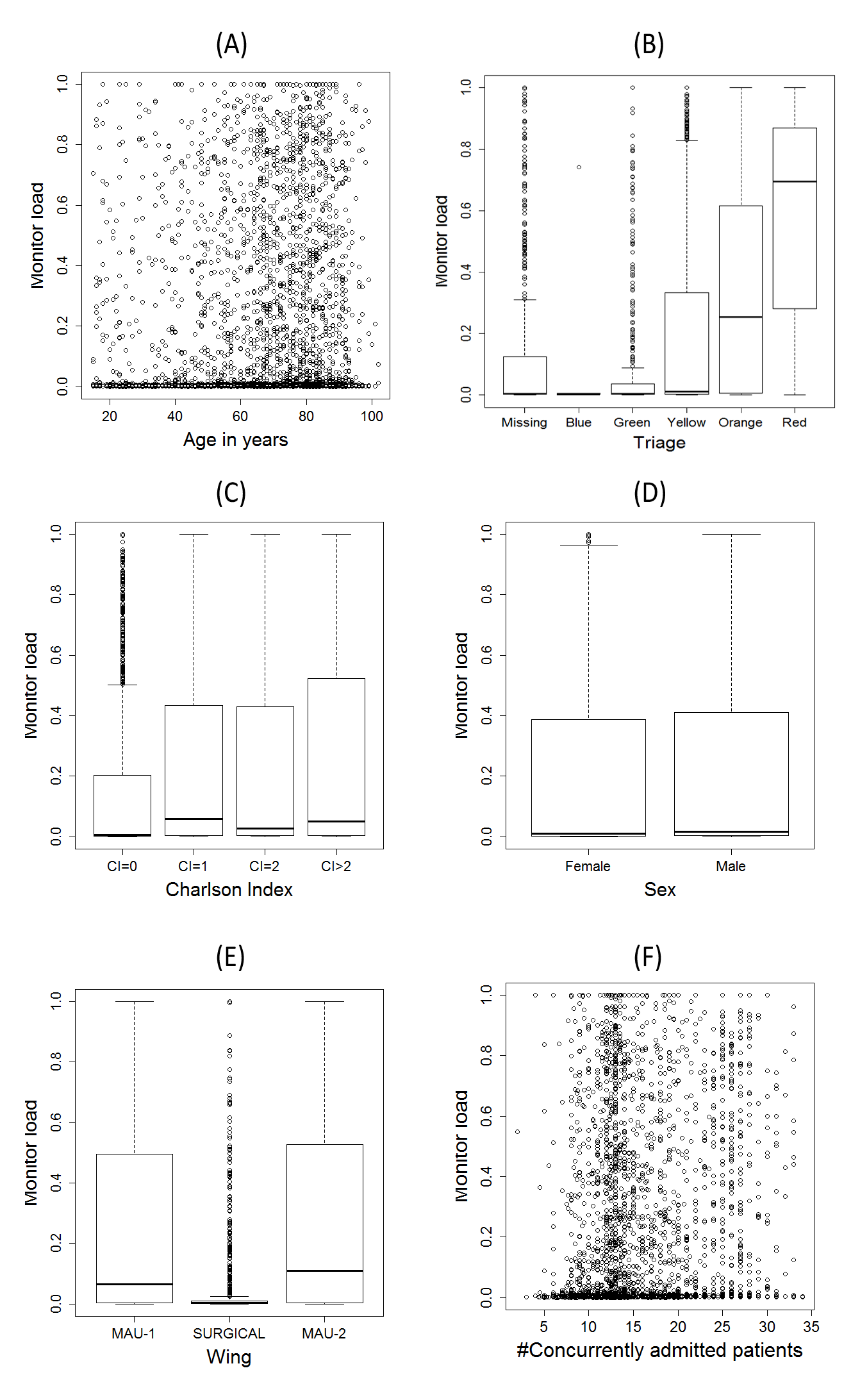

Supplement: Supplementary file 1 — Univariate plots of the relationship between monitor load and: (A) Patient age, (B) Triage (severity), (C) Charlson comorbidity index, (D) Sex, (E) Wings, (F) Number of concurrent patients admitted to the wing during each admission (TIFF 8610 kb) [file 10877_2016_9876_MOESM1_ESM.tif]
