# Supplementary material for: Factors related to monitoring during admission of acute patients
Source: J Clin Monit Comput. 2016 Apr 12;31(3):641–9. doi: 10.1007/s10877-016-9876-y (PMC5403848; doi:10.1007/s10877-016-9876-y)
Supplement: Supplementary file 2 — Regression results (DOCX 33 kb) [file 10877_2016_9876_MOESM2_ESM.docx]

**ONLINE SUPPLEMENT 2**

**Table 3. Regression results.**

| **Model** | **Variable** | **Coefficient** | **SE** | **t value** | **p value** | | **Holm-Bonferroni adjustment** | |
| --- | --- | --- | --- | --- | --- | --- | --- | --- |
| Multiple Ordinary Least Squares regression | | | | | | | | |
|  | (Intercept) | 0.21024 | 0.029829 | 7.048 | 0.0000 | *** |  |  |
|  | Distance | -0.0472082 | 0.104799 | -0.45 | 0.65241 |  |  |  |
|  | Triage Blue | -0.0821695 | 0.01832 | -4.485 | 0.0000 | *** |  |  |
|  | Triage Green | -0.0161513 | 0.015375 | -1.051 | 0.29356 |  |  |  |
|  | Triage Yellow | 0.0971611 | 0.016753 | 5.8 | 0.0000 | *** |  |  |
|  | Triage Orange | 0.3349287 | 0.039959 | 8.382 | 0.0000 | *** |  |  |
|  | Triage Red | -0.004093 | 0.000579 | -7.065 | 0.0000 | *** |  |  |
|  | North Wing | -0.1128428 | 0.015681 | -7.196 | 0.0000 | *** |  |  |
|  | East Wing | 0.0158118 | 0.011504 | 1.374 | 0.16941 |  |  |  |
|  | Concurrent Load | 0.0014378 | 0.000891 | 1.614 | 0.10657 |  |  |  |
|  | CI factor B | 0.0424236 | 0.014048 | 3.02 | 0.00255 | ** |  |  |
|  | CI factor C | 0.0342659 | 0.015715 | 2.18 | 0.0293 | * |  |  |
|  | CI factor D | 0.0537602 | 0.013504 | 3.981 | 0.0000 | *** |  |  |
|  | Age | 0.000544 | 0.000273 | 1.99 | 0.04663 | * |  |  |
|  | Male | 0.0052393 | 0.009881 | 0.53 | 0.596 |  |  |  |
| Multiple Quantile Regression | | | | | | | | |
| QR-10 | (Intercept) | 0.00174 | 0.00016 | 10.64796 | 0.0000 | *** | 0.0000 | *** |
|  | Distance | -0.00002 | 0 | -5.56269 | 0.0000 | *** | 0.0000 | *** |
|  | Triage Blue | -0.00015 | 0.00015 | -1.0361 | 0.3002 |  | 1.5012 |  |
|  | Triage Green | -0.00026 | 0.00006 | -4.26982 | 0.0000 | *** | 0.0001 | *** |
|  | Triage Yellow | 0 | 0.00008 | -0.01234 | 0.9902 |  | 4.9508 |  |
|  | Triage Orange | 0.00053 | 0.00016 | 3.20442 | 0.0014 | ** | 0.0069 | ** |
|  | Triage Red | 0.00282 | 0.01299 | 0.21737 | 0.8279 |  | 4.1397 |  |
|  | North Wing | -0.00047 | 0.0001 | -4.50336 | 0.0000 | *** | 0.0001 | *** |
|  | East Wing | -0.00003 | 0.00011 | -0.24215 | 0.8087 |  | 4.0434 |  |
|  | Concurrent Load | 0 | 0.00001 | 0.19616 | 0.8445 |  | 4.2225 |  |
|  | CI factor B | 0.00017 | 0.00013 | 1.35784 | 0.1746 |  | 0.8731 |  |
|  | CI factor C | 0.00035 | 0.00014 | 2.52054 | 0.0118 | * | 0.0589 |  |
|  | CI factor D | 0.00021 | 0.00008 | 2.593 | 0.0096 | ** | 0.0478 | * |
|  | Age | 0 | 0 | 1.86561 | 0.0622 |  | 0.3110 |  |
|  | Male | 0.00007 | 0.00006 | 1.1447 | 0.2524 |  | 1.2621 |  |
| QR-25 | (Intercept) | 0.00232 | 0.0003 | 7.64011 | 0.0000 | *** | 0.0000 | *** |
|  | Distance | -0.00007 | 0.00001 | -8.78338 | 0.0000 | *** | 0.0000 | *** |
|  | Triage Blue | 0.00003 | 0.0016 | 0.018 | 0.9856 |  | 4.9282 |  |
|  | Triage Green | -0.00064 | 0.00013 | -4.88565 | 0.0000 | *** | 0.0000 | *** |
|  | Triage Yellow | 0.00043 | 0.00012 | 3.59164 | 0.0003 | *** | 0.0017 | ** |
|  | Triage Orange | 0.00291 | 0.00092 | 3.15664 | 0.0016 | ** | 0.0081 | ** |
|  | Triage Red | 0.27857 | 0.10554 | 2.63944 | 0.0084 | ** | 0.0418 | * |
|  | North Wing | -0.00074 | 0.00019 | -3.9382 | 0.0001 | *** | 0.0004 | *** |
|  | East Wing | 0.00016 | 0.00031 | 0.5325 | 0.5944 |  | 2.9721 |  |
|  | Concurrent Load | 0.00004 | 0.00001 | 2.86051 | 0.0043 | ** | 0.0213 | * |
|  | CI factor B | 0.00031 | 0.0002 | 1.55008 | 0.1212 |  | 0.6061 |  |
|  | CI factor C | 0.00031 | 0.00023 | 1.35136 | 0.1767 |  | 0.8834 |  |
|  | CI factor D | 0.00032 | 0.00021 | 1.51793 | 0.1291 |  | 0.6457 |  |
|  | Age | 0.00002 | 0 | 5.43993 | 0.0000 | *** | 0.0000 | *** |
|  | Male | 0.00028 | 0.00012 | 2.39245 | 0.0168 | * | 0.0840 |  |
| QR-50 | (Intercept) | 0.01722 | 0.01203 | 1.43143 | 0.1524 |  | 0.7621 |  |
|  | Distance | -0.00078 | 0.00036 | -2.15595 | 0.0312 | * | 0.1558 |  |
|  | Triage Blue | -0.0094 | 0.00494 | -1.90197 | 0.0573 |  | 0.2864 |  |
|  | Triage Green | -0.00714 | 0.00537 | -1.33039 | 0.1835 |  | 0.9175 |  |
|  | Triage Yellow | 0.00502 | 0.00681 | 0.7369 | 0.4612 |  | 2.3062 |  |
|  | Triage Orange | 0.22114 | 0.02203 | 10.04025 | 0.0000 | *** | 0.0000 | *** |
|  | Triage Red | 0.62036 | 0.06095 | 10.17893 | 0.0000 | *** | 0.0000 | *** |
|  | North Wing | -0.00714 | 0.0077 | -0.9271 | 0.3540 |  | 1.7698 |  |
|  | East Wing | 0.02225 | 0.01235 | 1.80168 | 0.0717 |  | 0.3585 |  |
|  | Concurrent Load | 0.00018 | 0.00051 | 0.34523 | 0.7299 |  | 3.6497 |  |
|  | CI factor B | 0.00843 | 0.00951 | 0.88644 | 0.3755 |  | 1.8773 |  |
|  | CI factor C | 0.00514 | 0.00917 | 0.56023 | 0.5754 |  | 2.8769 |  |
|  | CI factor D | 0.00666 | 0.00711 | 0.9369 | 0.3489 |  | 1.7444 |  |
|  | Age | 0.00012 | 0.00008 | 1.44047 | 0.1498 |  | 0.7492 |  |
|  | Male | 0.00392 | 0.00278 | 1.41075 | 0.1584 |  | 0.7921 |  |
| QR-75 | (Intercept) | 0.34351 | 0.03606 | 9.52569 | 0.0000 | *** | 0.0000 | *** |
|  | Distance | -0.00424 | 0.00034 | -12.57453 | 0.0000 | *** | 0.0000 | *** |
|  | Triage Blue | -0.03655 | 0.10021 | -0.36476 | 0.7153 |  | 3.5766 |  |
|  | Triage Green | -0.02564 | 0.01597 | -1.60501 | 0.1086 |  | 0.5430 |  |
|  | Triage Yellow | 0.03319 | 0.01205 | 2.75515 | 0.0059 | ** | 0.0295 | * |
|  | Triage Orange | 0.24519 | 0.01354 | 18.11487 | 0.0000 | *** | 0.0000 | *** |
|  | Triage Red | 0.48847 | 0.03824 | 12.77515 | 0.0000 | *** | 0.0000 | *** |
|  | North Wing | -0.24076 | 0.02449 | -9.82993 | 0.0000 | *** | 0.0000 | *** |
|  | East Wing | 0.04678 | 0.02993 | 1.56317 | 0.1181 |  | 0.5906 |  |
|  | Concurrent Load | 0.00089 | 0.00123 | 0.72577 | 0.4680 |  | 2.3402 |  |
|  | CI factor B | 0.04612 | 0.01087 | 4.24414 | 0.0000 | *** | 0.0001 | *** |
|  | CI factor C | 0.04227 | 0.0125 | 3.38308 | 0.0007 | *** | 0.0037 | ** |
|  | CI factor D | 0.07279 | 0.01519 | 4.79261 | 0.0000 | *** | 0.0000 | *** |
|  | Age | 0.00023 | 0.00021 | 1.08998 | 0.2758 |  | 1.3791 |  |
|  | Male | 0.0053 | 0.00866 | 0.61204 | 0.5406 |  | 2.7028 |  |
| QR-90 | (Intercept) | 0.69091 | 0.06374 | 10.83897 | 0.0000 |  | 0.0000 | *** |
|  | Distance | -0.00507 | 0.00091 | -5.59623 | 0.0000 | *** | 0.0000 | *** |
|  | Triage Blue | -0.09486 | 0.06295 | -1.50687 | 0.1320 |  | 0.6598 |  |
|  | Triage Green | -0.10885 | 0.04923 | -2.21121 | 0.0271 | ** | 0.1355 |  |
|  | Triage Yellow | -0.00168 | 0.05048 | -0.03329 | 0.9735 |  | 4.8673 |  |
|  | Triage Orange | 0.09311 | 0.05032 | 1.85044 | 0.0643 |  | 0.3217 |  |
|  | Triage Red | 0.22728 | 0.05157 | 4.40763 | 0.0000 | *** | 0.0001 | *** |
|  | North Wing | -0.44805 | 0.02511 | -17.84156 | 0.0000 | *** | 0.0000 | *** |
|  | East Wing | 0.00905 | 0.01917 | 0.47214 | 0.6369 |  | 3.1843 |  |
|  | Concurrent Load | 0.00287 | 0.00124 | 2.30945 | 0.0210 | * | 0.1049 |  |
|  | CI factor B | 0.05352 | 0.02842 | 1.88293 | 0.0598 |  | 0.2990 |  |
|  | CI factor C | 0.04089 | 0.02871 | 1.42424 | 0.1545 |  | 0.7724 |  |
|  | CI factor D | 0.07614 | 0.01823 | 4.17551 | 0.0000 | *** | 0.0002 | *** |
|  | Age | 0.00058 | 0.00044 | 1.31444 | 0.1888 |  | 0.9440 |  |
|  | Male | 0.00035 | 0.01494 | 0.0231 | 0.9816 |  | 4.9079 |  |
