# Supplementary material for: Factors related to monitoring during admission of acute patients
Source: J Clin Monit Comput. 2016 Apr 12;31(3):641–9. doi: 10.1007/s10877-016-9876-y (PMC5403848; doi:10.1007/s10877-016-9876-y)
Supplement: Supplementary file 3 — Guide to interpretation of quantile regression results (DOCX 14 kb) [file 10877_2016_9876_MOESM3_ESM.docx]

**ONLINE SUPPLEMENT 3**

**Interpretation of quantile regression results**

Interpreting the regression coefficients in QR is a bit different from the approach of least squares regression, with the twist that it is more correct to view the exposure coefficients as indicators of gap performances on the response variable based on differences in exposures.

The intercept slope in Figure 3 yields the average monitor load for each quantile when controlling for all exposures, and is typical for QR as the regression analysis is conditional on the score of the response variable. Higher quantiles are naturally associated with higher response values, and thus the intercept increases accordingly [18].

As an example of how to read Figure 3 and Table 3, we see that for distance from nursing office, patients in the lower quartile are affected very little by distance, but as patients are monitored more, the influence from distance becomes increasingly negative. Patients in the 50^th^ quartile has a distance coefficient of -0.00078 and a intercept of 0.01722; meaning that patients in the median range of the sample on average have a monitor load of 0.01722, and the expected gap between average distance, and 1 SD above negatively affects monitoring load by -0.00078 when controlling for other exposures (Table 3). For the 90^th^ quartile, the intercept is 0.69091 and the gap between average distance (18.5 meters) and a 1 SD distance deviation (9.4 meters) is -0.00507. Hence, as monitor load increases, distance from nursing office has an increasingly negative effect. For patients above the 70^th^ quartile, the negative effect of distance does not increase anymore, but is still at its strongest when compared to the other quartiles.
